# Influence of the Mediterranean and Ketogenic Diets on Cognitive Status and Decline: A Narrative Review

**DOI:** 10.3390/nu12041019

**Published:** 2020-04-08

**Authors:** Federica Vinciguerra, Marco Graziano, Maria Hagnäs, Lucia Frittitta, Andrea Tumminia

**Affiliations:** 1 Endocrinology, Department of Clinical and Experimental Medicine, University of Catania, Garibaldi Medical Center, Via Palermo 636, 95122 Catania, Italy; vinciguerrafederica@gmail.com (F.V.); graziano.marco91@gmail.com (M.G.); maria.hagnas@rovaniemi.fi (M.H.); lfritti@unict.it (L.F.); 2 Center for Life Course Health Research, University of Oulu, Aapistie 5/PO Box 5000, 90014 Oulu, Finland; 3 Rovaniemi Health Center, Koskikatu 25, 96200 Rovaniemi, Finland; 4 Diabetes, Obesity and Dietetic Center, Garibaldi Medical Center, Via Palermo 636, 95122 Catania, Italy

**Keywords:** Alzheimer disease, cognition disorders, diet, Mediterranean, diet, ketogenic

## Abstract

Alzheimer’s disease (AD) is the most common form of senile dementia, accounting for up to 70% of dementia cases. AD is a slowly progressive disease, which causes global mental deterioration by affecting various cognitive areas. A growing body of evidence has demonstrated that lifestyle habits and nutritional patterns could delay the natural course of the neurodegeneration process. There is no single dietary pattern unequivocally proven to prevent AD. Nevertheless, epidemiological data suggest that by adopting several dietary habits, especially if accompanied with a healthy lifestyle, the negative consequences of AD could potentially be delayed. Alongside with others, two specific eating patterns have been well investigated concerning their potential beneficial effect on cognitive status: the Mediterranean diet (MedDi) and the Ketogenic Diet (KD). Despite the different underlying mechanisms, both of them have demonstrated a fairly profitable role in reducing or delaying cognitive impairment. The aim of the present narrative review is to overview the existing research on the efficacy of MedDi and KD against AD-related cognitive decline, focusing on the proposed protective mechanisms of action. Although the current knowledge on this complex topic does not allow us, at this point, to make exhaustive conclusions, this information could be of help in order to better characterize the possible role of MedDi and KD as nonpharmacological therapies in the treatment of AD and, more generically, of neurodegenerative disorders.

## 1. Introduction

Neurological diseases represent the largest cause of disability and the second leading cause of death worldwide [1]. A consistent number of these conditions are represented by neurodegenerative disorders, which determine a progressive and global deterioration of the cognitive functions such as memory, language, learning, comprehension, orientation, and judgment, thereby affecting people’s everyday life [2]. The prevalence of neurodegenerative disorders is growing at an alarming rate all over the world, largely due to the aging of population [3]. It has in fact been estimated that dementia rate will triplicate within 2050, reaching 135 million people suffering from these conditions globally [4,5].

Alzheimer’s disease (AD), the most common cause of dementia [6], is characterized by the accumulation of amyloid β (Aβ) and hyper-phosphorylation of the Tau protein, with additional reduced acetylcholine levels and cerebral blood flow [2,7]. Furthermore, modifications in the blood-brain barrier (BBB), oxidative stress, mitochondrial impairment, neuroinflammation, and alterations in the insulin signaling have been observed in this condition [8].

Treatment for AD can be divided into pharmacological and nonpharmacological strategies. Pharmacological treatments comprise the symptomatic therapies, e.g., acetylcholinesterase inhibitors or N-methyl-D-aspartate (NMDA) receptor antagonists [9] and “disease-modifying” therapies, which are directed towards amyloid accumulation or Tau protein hyper-phosphorylation [10,11,12]. Despite the multifaceted pharmacological approach, to date the effectiveness of pharmacologic treatments is still inconsistent and the curative treatment towards AD does not exist [13,14].

Nonpharmacological treatments are directed to prevent the neurodegeneration and additionally to modify the risk factors for AD [15,16]. There is, in fact, a growing body of evidence showing that healthy lifestyle habits play an important role in preventing or delaying cognitive impairment [17,18]. Thus, nutrition offers an interesting opportunity for investigation. Data from recent studies show that reducing the intake of trans-fat and saturated fats, (e.g., red and processed meats); decreasing the consumption of dairy products; and increasing intake of fruits, vegetables, legumes, and whole grains could be associated with the improvement of cognitive function and prevention of AD [19,20]. Moreover, it has been suggested that different eating patterns, including Mediterranean diet (MedDi), Ketogenic Diet (KD), caloric restriction, Dietary Approaches to Stop Hypertension (DASH), and Mediterranean-DASH diet Intervention for Neurological Delay (MIND), could possibly reduce the pathophysiological hallmarks of AD [19,21,22,23].

Among others, in the last decade, the MedDi and KD have been the two most studied eating patterns for their possible beneficial effect on cognitive functions. Both of them have, in fact, demonstrated to be effective in reducing or delaying cognitive impairment in older people by acting through different pathophysiological mechanisms [22,23,24]. Table 1 synthetizes the most important features of these two dietary patterns. The main characteristics of the MedDi is the high consumption of olive oil, legumes, whole grains, fruit, vegetables, nuts, poultry, fish, low-fat milk derivatives, moderate consumption of wine, and a reduced consumption of red meat, sausages, and refined cereal products [20]. Instead, KD is a very high-fat and low-carbohydrate diet, which forces the system to shift from glucose metabolism toward the metabolism of fatty acids with the production of ketone bodies [25].

Despite the increasing availability of preclinical and clinical scientific evidence regarding the nonpharmacological approach in the prevention of neurodegeneration, a review paper describing the current knowledge, differences, and similarities of underlying mechanisms and the exerted effects of these two nutritional approaches on cognitive function is still missing [22]. Another open issue regards the impact of these dietary patterns on specific populations characterized by clinical frailty, which have been demonstrated to be at higher risk to develop AD-related cognitive impairment, such as those individuals with diabetes [26], chronic kidney disease [27], and other chronic-degenerative disorders associated with the aging process [28,29]. These populations could, in fact, easily develop malnutrition and/or sarcopenia, which must be accurately avoided in order to prevent further cognition deterioration.

The aim of this narrative bibliographic review is to overview the existing literature on the efficacy and pathways through which MedDi and KD associate with age-related cognitive decline and, therefore, to evaluate their possible role in the prevention and/or treatment of neurodegenerative disorders.

## 2. Methodology

We analyzed several studies of the last 20 years (2000–2020) about the relationship between the MedDi/KD and the prevalence of AD-related cognitive impairment.

The revision started with a research on PubMed/MEDLINE of a series of keywords related to both the studied dietary patterns (either MedDi or KD) and their influence on cognitive functions (“Mediterranean diet”, “Ketogenic diet”, “Dietary patterns”, “Nutritional patterns”, “Alzheimer’s disease”, “Mild cognitive impairment”, “Cognition disorders”, “Cognitive decline”, “Neurodegeneration”). The key words for each component of the research were linked using “or” as a Boolean function, and the results of the two sections were combined by using the “and” Boolean for further search. Titles and abstracts of retrieved studies were screened to select potentially relevant articles. Full texts were analyzed independently to determine whether they met the established inclusion criteria. Moreover, references of eligible articles were searched manually for additional papers that could have been missed by the electronic search only.

With respect to article types, prospective cohort studies, retrospective and cross-sectional studies, randomized controlled trials, meta-analyses, and systematic and narrative reviews were included in the present work. Case reports and case series were also considered in the analysis when it was appropriate for completeness purposes. Finally, existing editorials and commentaries to the most relevant articles were comprised.

Afterwards, we included in the examination both the papers describing a significant direct or inverse relationship between the studied dietary pattern and the cognitive effects and those papers without statistically significant findings. We marked out the most important results for each study, focusing on the supposed underlying mechanisms through which the reported effect was determined.

Conversely, nonoriginal articles and studies in languages other than English were excluded.

The last update of our dataset was performed in March 2020. Considering the nature of the study, Ethics committee approval was not required.

## 3. Mediterranean Diet

The traditional MedDi is a nutritional pattern inspired by the food traditions of the populations that live in countries bathed by the Mediterranean Sea [30]. Among other benefits, MedDi has been linked to a lower risk of various chronic diseases [31], and it is assumed that its healthful effects are due to the high intake of monounsaturated fats (MUFAs) and polyphenols from olive oil, the high consumption of polyunsaturated fats (PUFAs) from fish, and the antioxidant properties of vegetables, fruits, and wine [32].

Several randomized controlled trials (RCTs) have examined the association between MedDi and cognitive function [33,34,35]. The Spanish PREDIMED (Prevención con Dieta Mediterranea) study was the cornerstone trial showing that patients at high cardiovascular risk consuming MedDi (supplemented with either extra-virgin olive oil or nuts) had a reduced incidence of cardiovascular events compared to those following a low-fat diet after a follow-up period of five years [36,37]. In a subcohort of 447 subjects (aged 55–80 years) participating in the PREDIMED study, an improved cognitive function was observed in the MedDi group compared to the group with low-fat dietary pattern, after a follow-up of 6.5 years [38,39].

Another RCT, the MedLey study, was conducted among 137 non-Mediterranean patients with a mean age of 72 years, who were randomly assigned to either a MedDi or a control diet (their usual diet). No significant beneficial effects during six months of study period were observed on cognitive function in the MedDi group compared to the control group. The limited number of participants, the short duration of follow-up (6 months)—especially for detecting modification of cognitive functions—and the milder modification of the nutritional habits of the patients in respect of their usual diet (intervention diet was essentially based on Australian foods rather than the conventional MedDi) could have effected on the nonobserved effects on cognitive function of MedDiet intervention group [34].

A third RCT regarding MedDi and cognitive performance was recently conducted among 65–79 year-old patients from Mediterranean and non-Mediterranean regions (the NU-AGE trial) [35]. In this study, the intervention group followed a MedDi-like diet, tailored on the cultural habits and dietary recommendations of the different regions involved in the study, while the participants of the control group followed their habitual diet. After one year of follow-up, individuals of the intervention group showed improvements in their cognitive performance compared to those of the control group, even if these differences were not statistically significant. Nevertheless, a higher adherence to the MedDi-like diet was related to a significant improvement of episodic memory, which is associated to neurodegeneration [35,40].

Moreover, a recent meta-analysis of RCTs showed an association of MedDi with an improved global cognition among healthy older adults when compared with controls [41]. Several other meta-analyses have been published regarding the role of MedDi on the process of neurodegeneration, suggesting an inverse association between adherence to MedDi and the risk of developing cognitive impairment [42,43,44].

A number of cross-sectional and longitudinal studies performed both in Mediterranean and non-Mediterranean countries and using different MedDi adherence scores have demonstrated that higher adherence to the MedDi is associated with a significantly lower risk for developing mild cognitive impairment (MCI) and AD [45,46,47,48,49]. Better cognitive functions and lower rates of cognitive decline have been also demonstrated in systematic reviews of RCTs and observational studies [32,50], concluding that a higher level of adherence to the MedDi nutritional pattern is associated with overall better cognitive performance. Additionally, adherence to MedDi has been associated with reduced mortality rates in AD patients [51].

Beside these observed positive associations of MedDi with the cognitive function, there are also some prospective studies that have not found association of MedDi with the improvement of cognitive status [52]. Nonobserved association was also seen in a Swedish longitudinal cohort study, with 12 years of follow-up, where the MedDi was not associated with the prevention of AD [53].

Several factors should be taken into account when interpreting these nonobserved associations. Firstly, the use of different score-based methods for evaluating the adherence to the studied nutritional pattern (e.g., the Trichopoulou’s or the Panagiotakos’s scores) [54,55] does not allow a homogeneous interpretation of the clinical findings. These scores have been mainly tested on Mediterranean cohorts and, thus, are not fully applicable to people living in non-Mediterranean countries and, therefore, having different dietary/lifestyle habits [47,56]. Secondly, it is important to consider that the abovementioned studies imply profound differences in terms of study design, average age of the study population, neuropsychological tests used, as well as the duration of the follow-up period. Nevertheless, the majority of the evidence suggests that MedDi has been shown to associate with improved cognitive function among elderly participants (Table 2).

As it has been stated by several authors, the MedDi cannot be merely considered only as a nutritional pattern. Rather, it represents a cultural heritage and a lifestyle, which is often associated with factors that are, per se, related to a better cognitive function, such as moderate physical activity and behaviors of social and familiar aggregation—that is, for example, eating meals together with friends or family members [30,69].

### 3.1. Mediterranean Diet and Cognitive Impairment in Type 2 Diabetic Patients

It is known that type 2 diabetes (T2D) is a risk factor for cognitive disorders, given that more than 30% of patients with T2D have MCI [70]. Diabetes has been shown to significantly deteriorate the cognitive function, not only inducing acute and chronic vascular disorders, but also increasing the risk for neurodegenerative diseases such as AD [71]. To date, there is a lack of scientific evidence regarding the role of MedDi on cognitive status and decline in T2D patients. However, one recent study investigated the correlation of two-year change in global cognitive status z-score, executive cognitive functions, and several cognitive tests to the adherence to different eating patterns (e.g., MedDi, DASH diet, Healthy Eating and Alternate Healthy Eating indices). Among others, adherence to MedDi was significantly associated with higher two-year change in global cognitive function in adults with T2D, thus demonstrating a positive effect of this eating pattern in people with impaired glucose metabolism [72]. Moreover, the study demonstrated that good glycemic control further sustained these benefits. Better cognitive scores were, in fact, confirmed in those patients under glycemic control at baseline and stable/improved over the two years of follow-up, but not for individuals with uncontrolled or poor/declined glycemic control at the end of the observation period. Therefore, the treatment of diabetes should be strictly targeted and effective in order to preserve optimal cognitive function. Further studies are needed to confirm these findings.

### 3.2. Suggested Mechanisms between Mediterranean Diet and Alzheimer’s Disease

To date, the pathways underlying the proposed neuroprotective effects of the MedDi are not fully understood. There are several possible mechanisms that could explain the effect of the nutritional pattern of MedDi on cognitive health. Neurodegenerative diseases such as AD are displayed with typical features of cognitive dysfunction, which may be partially corrected by adhering to a balanced nutritional pattern. Besides the histopathological hallmarks (e.g., Aβ accumulation, Tau hyper-phosphorylation), the neuronal loss seen in AD has been additionally associated with other mechanisms as reduced blood flow (due to the atherosclerotic process), mitochondrial dysfunction, oxidative stress, neuroinflammation, and alteration of the gut microbiota [32].

There is evidence that adherence to the MedDi may modify some of the major cardiovascular risk factors such as atherosclerotic dyslipidemia [36]. Furthermore, a meta-analysis of clinical trials, which compared the effects of the MedDi with low-fat diet on cardiovascular risk factors among 2650 study participants with and without T2D, confirmed that MedDi is more effective in reducing body weight, systolic and diastolic blood pressure, fasting blood glucose, and C-reactive protein during a two-year follow-up [73].

The benefits of the MedDi diet on the cognitive status are probably mediated by reduced oxidative stress and inflammation [20]. Evidence from epidemiological and clinical studies showed, in fact, that some of the main components of MedDi (e.g., fruit and vegetables) are able to reduce the brain damage induced by reactive oxygen species (ROS), by improving antioxidant defenses and by lowering lipid oxidation and platelet aggregation [74,75]. MUFAs, the most abundant types of fat in the MedDi, are able to increase antioxidant pathways, whereas saturated fatty acids (SFA) display a harmful effect. Higher plasma levels of lipoproteins rich in SFA rather than in MUFA could affect the responsiveness of low-density lipoprotein (LDL)-receptors in the liver, inducing an increase in LDL levels and making them more predisposed to oxidation, a condition that increases the formation of atherosclerotic plaques at vascular level [75,76].

Furthermore, some authors have proposed that MedDi could also influence Tau metabolism and Aβ plaque formation [77], however nonobserved associations have been also reported [78].

Additionally, mechanisms considering the association of MedDi with the modification of gut microbiota should be discussed. Significant reduction of several anti-inflammatory gut bacteria (e.g., Bacteroides fragilis) and increase of proinflammatory bacteria (e.g., Escherichia coli) have been, in fact, detected in patients with cognitive impairment and AD [79]. Several authors have demonstrated a positive correlation between MedDi and a beneficial modification of gut microbiota, with an increased amount of Bifidobacteria and, thereafter, increased anti-inflammatory and hypocholesterolemic activity [80].

**Figure 1:**
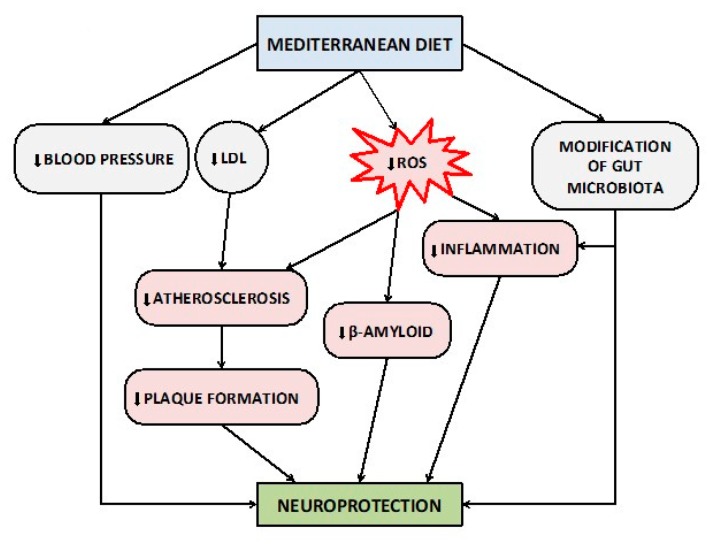
Proposed protective mechanisms of Mediterranean diet on brain health and cognitive functions. Abbreviations: LDL, Low-Density Lipoprotein; ROS, Reactive Oxygen Species.

The proposed protective pathways of MedDi on brain health are summarized in Figure 1. Although the scientific evidence on the role of the nutritional pattern of MedDi in the prevention and/or delay of cognitive impairment is continuously growing, the precise identification and description of these mechanisms requires even further preclinical and clinical studies to be fully elucidated.

## 4. Ketogenic Diet

The KD represents a particular dietary pattern capable of inducing and maintaining a chronic state of ketosis, that is, a metabolic condition where the body mainly uses ketone bodies (KBs) instead of glucose as the major fuel to produce energy [81]. The most powerful condition that can induce ketosis is fasting [82]. In fact, after less than 24 h of fasting, the body initiates the utilization of the majority of liver glycogen reserves and depends mainly on the endogenous synthesis of glucose and fatty acid oxidation to produce Adenosine Triphosphate (ATP) [82]. A similar process can be achieved with very low-carbohydrate diet and dietary patterns that imply a high percentage of energy intake derived from fatty acids [83].

In case of unavailability of glucose, the liver of an adult is able to release into the bloodstream per day up to 150–300 g of KBs [84], most of which are utilized by the tissues of high energy requirement (e.g., skeletal muscle, heart, and brain) [85]. In particular, in the brain, the mitochondria of the neuronal cells are able to oxidize the acetoacetic acid and the β-hydroxybutyric acid producing acetyl-CoA by supporting, in this way, the production of ATP via the Krebs cycle. This process becomes essential in the case of glucose deficiency, because the neurons are not able to carry out gluconeogenesis [86].

The neuroprotective role of ketosis is known since the 1920s, when it was observed among epileptic patients as a way to reduce the frequency of seizures, followed by prolonged fasting [86,87].

In 1921, Wilder et al. [88] proposed a protocol to utilize KD for the treatment of drug resistant epilepsy. In its original version, KD was able to provide a caloric intake reduced to about 75% of the energy requirement and included a limitation in the assumption of liquids. The induction of ketosis was implemented with a fasting period of 12–48 h followed by the gradual consumption of ketogenic meals [88].

The dietary restrictions for the induction and maintenance of ketosis have resulted in the search for alternative protocols. Some of these include the traditional induction of ketosis but without initial fasting, or a progressive reduction of carbohydrate content, which corresponds to a gradual increase in the lipid/carbohydrate ratio. The latter possibility of induction makes the start of the diet possible even in an outpatient setting. The replacement of a proportion of long-chain triglycerides, at the base of the classic protocol, with medium-chain triglycerides (MCTs), is a KD alternative introduced in 1971 by Huttenlocher et al. [89]. This alternative allows to increase the proportion of carbohydrates in the diet, since the MCTs are more ketogenic compared to long-chain triglycerides [90]. Another form of KD used for the treatment of drug-resistant epilepsy is a modified version called Atkins diet. It is based on an initial drastic reduction of carbohydrate intake, while the protein-containing foods can be consumed without restrictions. Subsequently, in the course of treatment, the carbohydrates are partially included. Although the Atkins diet needs less dietary control compared to the classical KD, it induces a low and fluctuating level of ketosis due to the neoglucogenic effect of the proteins [88,91,92,93].

The beneficial effects of the KBs on the human cognitive function have been observed in several studies. Two separate double-blinded clinical trials compared the influence of MCTs vs. placebo on cognitive capacity [94,95]. Both studies clearly showed that an increase in serum levels of β-hydroxybutyric acid was associated with an improvement of cognitive function and memory. Additionally, the blood KB levels were also positively associated with the improvement in verbal memory in patients with MCI even in the absence of differences in other cognitive functions [96]. In addition, some evidence received from Positron Emission Tomography imaging studies have shown an increase in brain uptake of KBs following administration of MCTs, suggesting that KBs could compensate for the glucose uptake deficiency that is present in the brain tissue of patients with AD [97,98]. The benefits of KDs have been demonstrated not only in adults but also in children, particularly in those with epilepsy, in which adherence to KD was associated with a better cognitive performance [99,100]. Table 3 summarizes both the animal and human studies, which aimed to characterize the role of KD on cognitive functions.

Evidence from preclinical studies reported positive effects of ketones on cognitive function [101,102] but also on the regulation of Aβ, which represents the main hallmark of AD. In a study using a mouse model of AD, Van der Auwera et al. [103] demonstrated for the first time a correlation between KD treatment and the reduction of Aβ expression and, consequently, of senile plaque formation and cerebral oxidative stress. This result was replicated in a subsequent study, where the authors showed that prolonged treatment with ketone esters were associated not only with a reduction of Aβ and Tau protein deposition, but also with the improvement of performance on learning and memory tests [104].

Furthermore, it has been demonstrated that ketones—blocking Aβ entry into neurons—reduce intracellular amyloid aggregation and improve mitochondrial function and memory ability [105].

In disagreement with these results, a study reported that KD improved motor performance but did not reduce the deposition of Aβ or Tau protein in a transgenic mouse model [106].

Zhao et al. [107] reported detrimental effects of KD on cognitive functions. In particular, the authors found that KD impaired spatial learning, memory, and brain growth in rats. It should be noted that the KD used in this trial had a fat-to-protein plus carbohydrate ratio that was more than 2-fold higher than standard KD diet and that protein represented only 8% of the diet, which is less than half of the protein content of a regular diet. Severe protein-energy restriction could be, therefore, the factor responsible for the reported negative effects on cognitive function and brain development [108].

In humans, some studies showed that administration of MCTs supplements, which raises beta-hydroxybutyrate (B-OHB) levels, improves cognition and memory in AD subjects in the short-term [109,110,111]. However, some of these studies suggest that neurological effects depend on the apolipoprotein E4 (ApoE4) genotype, the most prevalent genetic risk factor for AD, because cognitive benefits have been demonstrated only among subjects who were ApoE4-negative [109,110]. Nevertheless, a recent case study reported cognition improvements after KD in a patient who was heterozygous for ApoE4 and with a family history of AD and diagnosis of mild AD [112].

Administration of MCTs in patients with mild-to-moderate AD induces significant improvement in immediate and delayed logical memory tests after 8 weeks and in the digit-symbol coding test and immediate logical memory tests after 12 weeks [113]. A positive effect on memory function after KD has also been demonstrated in older adults with MCI [96,114].

The Ketogenic Diet Retention and Feasibility Trial conducted on 15 patients with AD showed that during ketosis, obtained with MCTs-supplemented KD, the mean of the Alzheimer’s disease Assessment Scale–cognitive subscale score (ADAS–cog) significantly improved but returned to baseline after 1-month washout period [115].

A recent pilot study evaluated neurological effects of a modified Mediterranean–ketogenic diet (MMKD) in older adults at risk for AD [116]. MMKD differed from KD because it provided slightly higher consumption of carbohydrate such as vegetables and fruits, proteins derived from fish, and fats derived from olive oil. In this trial, an increased cerebral perfusion and cerebral ketone body uptake following MMKD has been reported [116].

Despite abovementioned beneficial effects of KD on patients with MCI or AD, evidence from a randomized controlled study suggested that KD has no effect on the cognitive performance of 11 healthy, normal-weight individuals [117].

### 4.1. Suggested Mechanisms between Ketogenic Diet and Alzheimer’s Disease

In recent years, several hypotheses have been proposed to explain the neuroprotective role of KBs [23]. Some studies have shown that during a long period of starvation, KBs may provide up to 70% of cerebral energy requirements, representing a more efficient energy source than glucose due to the fact that KBs can enter directly into the Krebs cycle to produce ATP, while glucose needs to be first metabolized through glycolysis [118]. This effect seems to play a particularly important role in individuals with AD, because in this condition, there is an alteration of the BBB that results in altered expression of some membrane transporters and, in particular, a reduction of the expression of the glucose transporters. Under these conditions, the brain is more dependent from the catabolism of KBs that, unlike glucose, cross the BBB hanks to specific carriers that are not downregulated in AD [91]. This effect may explain why, in fasting patients suffering from MCI or AD, after ingestion of a dose of MCTs able to increase the plasma levels of β-hydroxybutyric acid, a significant improvement in the cognitive abilities was shown compared to placebo [109].

KBs improve mitochondrial function, by enhancing nicotinamide adenine dinucleotide (NADH) oxidation in the mitochondrial respiratory chain and by inhibiting mitochondrial permeability transition [119].

As already mentioned, oxidative stress and chronic inflammation represent two of the most important neurotoxic mechanisms underlying the development and progression of MCI and AD, as they cause neuronal death in the brain areas responsible for memory and cognitive processes [120].

The metabolism of KBs has been demonstrated to associate with a lower production of ROS, and thus with a reduction in oxidative stress, as compared to glycolysis, assuming an additional neuroprotective mechanism carried out by these compounds [23]. In particular, B-OHB reduces mitochondrial ROS production and inhibits histone deacetylases, upregulating the transcription of some genes that are protective against oxidative stress [121,122]. Moreover, KBs contribute to the reduction of ROS production through the expression of mitochondrial uncoupling protein (UCP), thereby decreasing mitochondrial membrane potential [123]. In addition, the activation of peroxisome proliferator-activated receptor gamma (PPARγ) after KD seems to inhibit neuroinflammation in mouse hippocampus [124]. In addition, it has been reported that KD has the capability to induce antioxidant glutathione peroxidase activity in the hippocampus with a potential protective effect against neurodegeneration of this structure [125,126].

Other neuroprotective mechanisms that have been highlighted against the development of AD are the reduction of the Amyloid Precursor Protein (APP) production [91] and of Aβ deposition [103]. Moreover, a recent study has shown that in vitro KBs are able to induce Aβ clearance from the brain to blood [127]. According to some evidence, treatment with KD has been also associated with an increase of angiogenesis and capillary density, suggesting that the KBs may support the cognitive processes through an improvement of the cerebral microvascularization [94].

**Figure 2:**
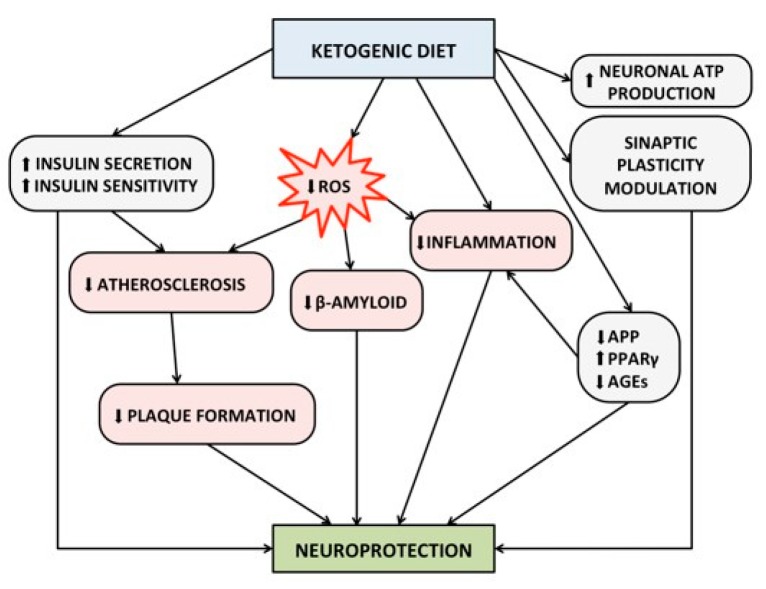
Proposed protective mechanism of ketogenic diet on brain health and cognitive functions. Abbreviations: ATP, Adenosine Triphosphate; AGEs, Advanced Glycosylated End-products; APP, Amyloid Precursor Protein; PPAR-γ, Peroxisome Proliferator-Activated Receptor gamma; ROS, Reactive Oxygen Species.

Finally, as is widely known, the KD improves the ability to secrete insulin by the β-cell as well as enhances insulin-sensitivity [128]. Insulin resistance is a major risk factor for the development and progression of cognitive impairment and AD, because it is associated with increased cerebral atherosclerosis, smaller volume of the hippocampus, formation of advanced glycation end-products (AGEs), increased oxidative stress, production of proinflammatory cytokines, and deposition of beta-amyloid [97]. Insulin resistance has been also demonstrated in the brain in patients with AD, even in the absence of diabetes mellitus [129]. In the brain, insulin exerts a neuroprotective effect by modulating synaptic plasticity, memory functions, and learning [130]. In particular, the hippocampal neurons express the glucose transporter type 4 (GLUT–4) which, unlike the GLUT–1 and GLUT–3 (that are ubiquitously expressed at the neuronal level), needs the presence of insulin in order to allow the entry of glucose in the cell [131]. Figure 2 summarizes the underlined protective mechanisms of KD against cognitive impairment.

For the abovementioned characteristics, KD appears to be a promising option for slowing the progression of cognitive impairment in patients with MCI and AD. However, the best results on memory and learning ability can be achieved when the ketosis status is induced at the earliest stages of cognitive impairment.

### 4.2. Ketogenic Diet in the Elderly Patients, Possible Concerns

Even if the effects of KD on cognition function seem encouraging, there are several issues raised by the utilization of this nutritional pattern in people who suffer from neurological disorders. Furthermore, among frail elderly individuals, the comorbidity is common, and they are at high risk for developing malnutrition.

One of the characteristics of KD is the reduction of appetite, which may be additionally associated with gastrointestinal side effects [132]. This anorectic effect could further reduce dietary portions and overall food intake in elderly patients following a KD eating pattern with the subsequent increased risk of malnutrition and deterioration of neurological symptoms [25].

Moreover, since the risk of sarcopenia is high in the elderly population, we have to consider that, although the percentage of energy intake belonging from proteins is often normal or even higher than recommended, KD may lead to an insufficient supply of protein, leading to catabolism of muscular proteins [133].

Lastly, because of the concerns regarding use of KD in people with T2D, chronic kidney disease, and disordered eating patterns, further research is needed before recommendations can be made for these subgroups of individuals. Adopting a KD eating plan can, in fact, increase diuresis and rapidly reduce plasma glucose-inducing hypoglycemia.

Therefore, consultation with a specialist is imperative prior to the consumption of KD to avoid dehydration, and to modify the dosages of antihyperglycemic medications and insulin, if necessary, in order to prevent hypoglycemia in people with T2D [134].

## 5. Discussion

Acceleration of aging worldwide, and thus increasing incidence of neurodegenerative diseases such as AD, in combination with the fact that no effective drugs for the treatment of dementia has been discovered so far, have increased interest in the identification of possible modifiable factors that could prevent, or at least delay, the development of cognitive impairment in the elderly. In this regard, during the last two decades, a growing body of evidence has been produced in order to elucidate the role of healthy lifestyle in counteracting the negative consequences of the aging process on cognitive health. In this regard, proper nutrition has been demonstrated to be an important factor for the maintenance of good cognitive function among healthy individuals and for the prevention of further mental deterioration in patients already affected by cognitive impairment. Furthermore, for a person with AD or dementia, poor nutrition may worsen behavioral symptoms and cause excessive weight loss.

With this review, we aimed to overview the current knowledge on the links between two of the most used eating patterns (e.g., MedDi and KD) and the risk of developing mild-to-severe cognitive damage, focusing specifically on the supposed underlying pathophysiological mechanisms. Our effort was directed to comprehensively show, in an orderly and equable fashion, a large amount of studies which are, so far, unbalanced between the two studied nutritional patterns—the evidence on MedDi being much wider.

The strength of the present narrative review consists in its original approach, aiming to highlight differences and similarities of these two dietary patterns as well as to stress both the common and distinct molecular pathways related to cognitive function. Secondly, despite our study consisting of a bibliographic and nonsystematic review, it used broad inclusion criteria aimed at developing a comprehensive and complete idea about the effect of both diets on cognition. Thirdly, unlike several previously published works) [135,136,137], it considered both dietary patterns holistically rather than the association between specific macronutrient or micronutrient intakes and cognitive function (in terms of improvement or deterioration). We believe that such an approach could be more suggestive of a wider and more complete message on the efficacy of the whole dietary approach rather than of single foods or supplements.

This review also has some limitations that might have affected the reporting of our results. Among them, it should be mentioned that dietary assessment methods varied from one study to the other, and this might have an effect on the outcomes and conclusions of the studies. More generically, the substantial problem in using the review approach for analyzing correlations and biological mechanisms is the heterogeneity in methodology, population, and outcome measurements between the retrieved studies.

Several issues must be considered in order to correctly interpret the reported literature in a meaningful way. By revising current scientific literature, in fact, we realized that overall evidence is not strong enough to show that any type of nutritional approach can significantly reduce the incidence of AD or other neurodegenerative diseases [24,138,139]. The reasons why we cannot, at this point, make exhaustive conclusions on this regard are multifaceted.

One issue is that most studies on the effects of diet on dementia are based on dietary questionnaires completed by participants who already may have trouble recalling what they ate or have memory problems [140].

Additionally, it may be challenging to assess the efficacy of specific dietary patterns such as KD for an adequately long study period, considering certain constraints of the application of such diets in older people with concomitant illness.

Finally, we believe that making a choice between different and specific eating patterns could be an incorrect simplification for a complex problem, which instead should be handled with personalized approaches in order to address individual nutritional needs. Many researchers, in fact, speculate that making healthy food choices (and not following specific diets) may improve the modifiable risk factors for cognitive deterioration such as dyslipidemia, diabetes, atherosclerosis, which may in turn reduce the risk of MCI or AD.

## 6. Conclusions

Current scientific literature does not provide exhaustive conclusions on the impact of any dietary patterns such as MedDi and KD on neurodegenerative disorders like AD.

Nevertheless, several useful clinical messages emerge from the careful review of published papers and are summarized as follows: (1) Even if the vast majority of evidence supports MedDi as the nutritional pattern, which has been demonstrated to be fairly efficacious in the preservation of several cognitive functions, other nutritional patterns such as KD have also been associated with beneficial effects. (2) Although the consumption of KD has been very popular in the last few years, KD should be recommended only in specific cases and for a limited time, considering both the insufficient available data in human models (especially in the elderly) and the possible risks related to its prolonged use. (3) MedDi, independently from the effectiveness in reducing the risk of development and progression of cognitive impairment, has already been demonstrated to reduce risk for cardiovascular disease and mortality and can be recommended to patients—despite their age, clinical conditions, and comorbidities. Furthermore, it can be tailored on the majority of the cultural habits within the different countries and regions, and along with physical activity it represents an overall healthy lifestyle more than a simple dietary pattern.

More research and clinical trials are needed to determine to what degree a certain dietary pattern can be able to prevent AD or to slow its progression.

## Figures and Tables

**Table 1:** Main characteristics of the Mediterranean and ketogenic diet eating patterns.

Mediterranean Diet		Ketogenic Diet	
Characteristics: dietary pattern rich in monounsaturated fatty acids and polyphenols (mainly from olive oil), polyunsaturated fatty acids (from fatty fish), antioxidants, vitamins and minerals (magnesium, potassium, calcium, iodine, zinc, selenium). Poor in saturated fatty acids		Characteristics: very high-fat and low-carbohydrate diet, (carbohydrate intake ≤10% of consumed energy). This eating pattern forces the system to shift from glucose metabolism toward the metabolism of fatty acids with the consequent production of ketone bodies.	
Moderate to high consumption	Low consumption	High consumption	Avoided consumption
Whole grains Vegetables Fruits Olive oil Olives/nuts/seeds Low-fat dairies Herbs/spices/garlic/onions Eggs White meat Fish/seafood Potatoes Legumes Red wine	Salt Red meat Processed meat Sweets	Meat Fish and seafood High-fat dairies	Whole/refined grains Flour products Starchy vegetables Fruit juices Legumes Wines, beer, drinks with added sweeteners

**Table 2:** Evidence on association between Mediterranean diet and cognitive decline.

Randomized Controlled Trials (RCT)			
Study Names and Characteristics	Main Findings	Year	Reference
Nu-Age Study	Higher adherence to Mediterranean diet (MedDi) was related to a significant improvement in global cognition and episodic memory after 1 year in 1279 relatively healthy older adults.	2018	[35]
Medley Study	Adherence to MedDi compared to usual diet had no beneficial effects on cognitive performances (executive functioning, speed of processing, memory, visual-spatial ability, and overall age-related cognitive performance) in 137 healthy older adults (6 months RCT).	2016	[34]
Predimed Study	Mediterranean diet (MedDi) supplemented with olive oil or nuts and compared to low-fat diet is associated with improved cognitive function evaluated with neuropsychological tests among 447 cognitively healthy older women at high cardiovascular risk after 4.1 years.	2015	[38]
Predimed-Navarra Study	MedDi supplemented with olive oil or nuts compared to low-fat diet is associated with improved cognitive function examined by Mini-Mental State Examination (MMSE) and Clock Drawing Test (CDT) in 522 patients at high vascular risk after 6.5 years.	2013	[39]
**Reviews and Meta-Analyses**			
Meta-analysis (15 cohort studies and 2 RCTs)	Adherence to MedDi improves global cognition of healthy older adults in particular in terms of the following: delayed recall, working memory, processing speed, and reasoning.	2017	[41]
Systematic review and meta-analysis (9 cohort studies)	Higher adherence to the MedDi is inversely associated with the developing of cognitive disorders.	2017	[57]
Systematic review and meta-analysis (43 studies)	Higher adherence to MedDi and higher consumption of unsaturated fatty acids, antioxidants, and B vitamins decrease the risk of dementia.	2016	[58]
Systematic review (6 cross-sectional studies, 1 trial, 12 longitudinal studies, 3 meta-analyses)	Higher adherence to MedDi is associated with less cognitive decline, dementia, or AD, as shown by 4 of 6 cross-sectional studies, 6 of 12 longitudinal studies, 1 trial, and 3 meta-analyses.	2015	[59]
Systematic review and meta-analysis (5 studies)	Higher adherence to the MedDi is associated with a reduced risk of developing mild cognitive impairment (MCI) and Alzheimer’s disease (AD), and a reduced risk of progressing from MCI to AD.	2014	[42]
Systematic review (11 published articles)	Higher adherence to MedDi is associated with better cognitive function and lower rates of cognitive decline and AD.	2013	[50]
**Cross-Sectional Studies**			
Cross-sectional study	Higher adherence to MedDi was independently associated with better cognitive function and lower risk of cognitive impairment in 5907 community-dwelling older adults. Higher scores were independently associated with significantly better cognitive status in a dose-response manner.	2017	[60]
Cross-sectional study	MedDi adherence was positively associated with the MMSE score in elderly men (*n* = 237) but inversely associated in women (*n* = 320) residing in Velestino, Greece. Individual food groups or nutrients did not achieve a statistically significant association to MMSE score modifications.	2013	[61]
Cross-sectional study	AD and MCI patients had a lower adherence to the MedDi than healthy controls in Australian population (149 patients with AD, 98 with MCI, 723 healthy controls).	2012	[46]
Case-control study	Higher adherence to the MedDi was the main predictor of AD status in a case-control study nested within a community-based cohort in New York (194 patients with AD vs. 1790 nondemented subjects).	2006	[45]
**Longitudinal Studies**			
Prospective cohort study	During a mean follow-up of 12 years there was no association between MedDi-like diet adherence and the development of cognitive dysfunction among 1138 elderly Swedish men.	2015	[53]
Prospective Study	Long-term MedDi adherence was related to moderately better cognition, but not with cognitive change (16,058 women from the Nurses’ Health Study, aged 70 years or older, 6-years follow-up).	2013	[62]
Cache County Study on Memory, Health, and Aging	Higher adherence to MedDi was associated with higher levels of cognitive function in elderly men and women over an 11-year period (*n* = 3831 individuals aged ≥65 years).	2013	[63]
Regards (Reasons for Geographic and Racial Differences in Stroke) Study	Higher MedDi adherence was associated with lower incidence of cognitive impairment in 17,478 individuals (mean follow-up of 4 years).	2013	[64]
PATH Through Life study	Adherence to MedDi was not found to be protective against cognitive decline. Conversely, an excess of caloric intake and a higher consumption of monounsaturated fats were predictive of MCI (1528 participants; follow-up period of 4 years).	2012	[65]
Prospective cohort study	Higher MedDi adherence was associated with lower risk of incident MCI among 1233 nondemented individuals. The odds ratio of MCI was reduced with both high vegetable intake and high polyunsaturated fatty acid consumption.	2010	[66]
Prospective cohort study	Higher adherence to MedDi was associated with slower decline of MMSE but no other cognitive tests and was not associated to the risk of incident dementia (1410 adults aged 65 or older, 5 years follow-up).	2009	[52]
Longitudinal study	Higher adherence to the MedDi is associated with reduced risk of MCI, but also reduced risk of developing AD (1393 cognitively normal participants, mean follow-up of 4.5 years).	2009	[67]
Prospective cohort study	Higher adherence to the MedDi and higher physical activity were independently associated with reduced risk for AD in 1880 community-dwelling elders (mean follow-up 5.4 years).	2009	[49]
EPIC-Greece cohort (European Prospective Investigation into Cancer and Nutrition)	Adherence to the MedDi was not associated with MMSE score (732 individuals; follow-up period 6–13 years). Physical activity is a significant predictor of cognitive function in the elderly. Seed oil intake may adversely affect cognition.	2008	[68]
Community-based clinical trial	A total of 2258 nondemented individuals in New York were prospectively evaluated every 1.5 years. Higher adherence to the MedDi was found to be associated with lower risk for AD.	2006	[48]

**Table 3:** Evidence on association between ketogenic diet and cognitive decline.

Animal Studies			
Study characteristics	Main findings	Year	Reference
Murine model	Ketogenic Diet (KD) may be able to enhance cognitive functions.	2018	[101]
Murine model	KD improves memory in aging mice.	2017	[102]
Murine model	Ketones significantly suppress intracellular β-amyloid (Aβ) accumulation and improve learning and memory function in symptomatic murine model of AD.	2016	[105]
Murine model	A ketone ester diet improves performance on learning and memory tests and reduces the amounts of Aβ and phospho-Tau in the brain in a mouse model of AD.	2013	[104]
Murine model	KD enhances motor performance but not cognition and Aβ or Tau deposition in murine models of AD.	2013	[106]
Murine model	KD reduces total Aβ levels in a mouse model of Alzheimer’s (AD) after 43 days.	2005	[103]
Murine model	KD impaired visual–spatial learning, memory, and brain growth in immature rats	2004	[107]
**Clinical Studies**			
Pilot study	Modified KD is associated with increased cerebral perfusion and improvement of memory performance in older adults at risk for AD (*n* = 20 patients).	2020	[116]
Case report	KD improves cognitive assessment of a 71-year-old female, heterozygous for ApoE4 with a family history of AD and diagnosis of mild AD after 10 weeks.	2019	[112]
Clinical trial	KD had no effect on vigilance, visual learning, and memory, working memory, and executive function (*n* = 11 healthy participants).	2019	[117]
Clinical Study	KD improved immediate and delayed logical memory tests after 8 weeks and both digit-symbol coding test and immediate logical memory test after 12 weeks in 20 patients with mild-to-moderate AD.	2019	[113]
Case report	KD improves cognitive assessment of a 57-year-old female previously diagnosed with comorbid mild cognitive impairment (MCI) and metabolic syndrome.	2018	[114]
Single-arm pilot trial: Ketogenic Diet Retention and Feasibility Trial (KDRAFT)	KD supplemented with medium-chain triglyceride improves AD Assessment Scale-cognitive subscale (ADAS-cog) after 3 months.	2018	[115]
Case report	Hyperketonemia induced by beta-hydroxybutyrate (B-OHB)-promoting ketone monoester induces cognitive improvement	2015	[111]
Clinical trial	KD improves memory function in older adults with MCI (*n* = 23 participants).	2012	[96]
Clinical trial	Ketosis induced by oral daily administration of ketogenic compound AC-1202 determines a significant improvement in the Alzheimer’s Disease Assessment Scale-Cognitive Subscale (ADAS-Cog) in E4(-) AD patients.	2009	[110]
Clinical trial	Administration of medium-chain triglycerides facilitated cognitive performance on the Alzheimer’s Disease Assessment Scale-Cognitive Subscale (ADAS-cog) only in older adults with AD or MCI who were apolipoprotein E4(-) AD patients.	2004	[109]
